# Aflatoxins and fumonisins co-contamination effects on laying hens and use of mycotoxin detoxifiers as a mitigation strategy

**DOI:** 10.1007/s12550-024-00566-x

**Published:** 2024-10-15

**Authors:** Phillis E. Ochieng, David C. Kemboi, Sheila Okoth, Siegrid De Baere, Etienne Cavalier, Erastus Kang’ethe, Barbara Doupovec, James Gathumbi, Marie-Louise Scippo, Gunther Antonissen, Johanna F. Lindahl, Siska Croubels

**Affiliations:** 1 Department of Food Sciences, Laboratory of Food Analysis, Faculty of Veterinary Medicine, University of Liège, 4000 Liège, Belgium; 2 Department of Pathobiology, Pharmacology and Zoological Medicine, Laboratory of Pharmacology and Toxicology, Faculty of Veterinary Medicine, Ghent University, 9820 Merelbeke, Belgium; 3 Department of Animal Science, Chuka University, P.O. Box 109-60400, 00625 Chuka, Kenya; 4 Department of Biology, Faculty of Science and Technology, University of Nairobi, P.O. Box 30197-00100, Nairobi, Kenya; 5 Department of Clinical Chemistry, Center for Interdisciplinary Research On Medicines (CIRM), University of Liège, University Hospital of Liège, 4000 Liège, Belgium; 6 Consultant, 00100 Nairobi, Kenya; 7 dsm-firmenich Animal Nutrition and Health R&D Center Tulln, 3430 Tulln, Austria; 8 Department of Veterinary Pathology, Microbiology, and Parasitology, Faculty of Veterinary Medicine, University of Nairobi, P.O. Box 29053-00100, Nairobi, Kenya; 9 Chair Poultry Health Sciences, Faculty of Veterinary Medicine, Ghent University, 9820 Merelbeke, Belgium; 10 International Livestock Research Institute (ILRI), P.O. Box 30709-00100, Nairobi, Kenya; 11 Department of Medical Biochemistry and Microbiology, Uppsala University, 751 05 Uppsala, Sweden; 12 Department of Clinical Sciences, Swedish University of Agricultural Sciences, 750 07 Uppsala, Sweden

**Keywords:** Aflatoxins, Bentonite, Egg, Food and feed safety, Fumonisins, Kenya, Laying hen

## Abstract

This study examined the effects of fumonisins (FBs) and aflatoxin B1 (AFB1), alone or in combination, on the productivity and health of laying hens, as well as the transfer of aflatoxins (AFs) to chicken food products. The efficacy and safety of mycotoxin detoxifiers (bentonite and fumonisin esterase) to mitigate these effects were also assessed. Laying hens (400) were divided into 20 groups and fed a control, moderate (54.6 µg/kg feed) or high (546 µg/kg feed) AFB1 or FBs (7.9 mg/kg feed) added diets, either alone or in combination, with the mycotoxin detoxifiers added in selected diets. Productivity was evaluated by feed intake, egg weight, egg production, and feed conversion ratio whereas health was assessed by organ weights, blood biochemistry, and mortality. Aflatoxins residues in plasma, liver, muscle, and eggs were determined using UHPLC-MS/MS methods. A diet with AFB1 at a concentration of 546 µg/kg feed decreased egg production and various AFB1-contaminated diets increased serum uric acid levels and weights of liver, spleen, heart, and gizzard. Interactions between AFB1 and FBs significantly impacted spleen, heart, and gizzard weights as well as AFB1 residues in eggs. Maximum AFB1 residues of 0.64 µg/kg and aflatoxin M1 (below limits of quantification) were observed in liver, plasma, and eggs of layers fed diets with AFB1. The mycotoxin detoxifiers reduced effects of AFB1 and FBs on egg production, organ weights, blood biochemistry, and AFB1 residues in tissues. This study highlights the importance of mycotoxin detoxifiers as a mitigation strategy against mycotoxins in poultry production.

**Supplementary Information:**

The online version contains supplementary material available at 10.1007/s12550-024-00566-x.

## Introduction

Poultry requires less space than other livestock like cattle and is a major source of income for low-income populations in sub-Saharan Africa (SSA). The majority of the flock is made up of chickens, but pigeons, ducks, ostriches, turkeys, quails, and guinea fowls are also becoming more and more significant (Magothe et al. 2012). Commercial poultry farming in SSA remains unable to meet the region’s need for this protein source (Akinola & Essien 2011). A major obstacle facing commercial chicken rearing in SSA is the scarcity of reasonably priced and high-quality feed. Furthermore, the majority of small-scale farmers in the SSA nations are ignorant that animal health and productivity can be negatively impacted by poor quality feeds that contain mycotoxins, among other contaminants (FAO 2022).

Mycotoxins are secondary metabolites produced in storage by certain fungi such as *Aspergillus* and *Penicillium* or in the fields by *Fusarium* fungi. Although more than 400 mycotoxins have been found, aflatoxin B1 (AFB1), fumonisin B1 (FB1), zearalenone, deoxynivalenol (DON), T-2 toxin, and ochratoxin A (OTA) are the most significant mycotoxins in animal production and human health because of their widespread incidence and toxicities (Kemboi et al. 2020).

Aflatoxin B1-contaminated feeds have been associated with immunosuppression, stunted growth, impaired reproductive function that results in delayed age of maturity, decreased egg production and hatchability, and poor egg quality in layer chickens (Fernandez et al. 1994; Lee et al. 2012). Moreover, consumers of poultry products may be in danger due to the transferability of mycotoxins from feed to these products. Studies have reported the presence of AFB1 in liver, kidney, muscle, and eggs of chickens from markets or slaughterhouses (Iqbal et al. 2014; Sineque et al. 2017). Aflatoxins (AFs) were also found in tissues from chickens fed diets contaminated with AFs (Magnoli et al. 2017; Ochieng et al. 2023; Trucksess et al. 1983). Poultry is comparatively resistant to the toxicities of FBs although damages to the kidney, liver, and gastrointestinal tract have been documented (Antonissen et al. 2014; Chen et al. 2021). Fumonisins are not often found in poultry products and trace levels were detected in chicken tissues, blood, and eggs in recent investigations (Antonissen et al. 2020; Tangni et al. 2020; Tardieu et al. 2021).

Multiple mycotoxins can contaminate feeds either because several toxigenic fungi contaminate the same feed or because the same fungi produce multiple mycotoxins (Njobeh et al. 2012). According to a survey of mycotoxin contamination of chicken feeds and feed ingredients from SSA, co-occurrence of AFs and FBs was the most common type of multiple contamination (Ochieng et al. 2021). When compared to their individual effects, interactions between mycotoxins can make their effects more severe, even at low concentrations (Huff et al. 1986). Few studies have evaluated the effects of co-contamination with AFB1 and FB1 on the immune system, blood biochemistry, and organs of broiler chickens (Ochieng et al. 2023; Tessari et al. 2006, 2010).

Mycotoxin detoxifiers such as mycotoxin binders that bind to mycotoxins and prevent bloodstream absorption or mycotoxin modifiers that convert mycotoxins into less toxic compounds are considered sustainable post-harvest methods of protecting animals from the harmful effects of mycotoxins that are already in feed and being ingested by the animals. Popularly used mycotoxin binders are clay minerals and physicochemical qualities of these clays are influenced by various aspects, including their source and spacing within the layers, which ultimately determine their ability to adsorb mycotoxins (Rosa et al. 2001). Compared to natural clay, artificially modified clays exhibit greater interlayer spacing and therefore increased mycotoxin-sequestering potential (Laurain et al. 2021). Bentonite (BENT) is one of the clay minerals that has been used as a mycotoxin binder to reduce AFB1 toxicities (Pappas et al. 2016; Saminathan et al. 2018). In addition to bentonite clay, biological elements, including plant extracts, algae, and *Trichosporon mycotoxinivorans*, which also function as mycotoxin modifiers, were included in the mycotoxin binder utilized in this study (Mesgar et al. 2022). The mycotoxin modifier employed in this study is known as fumonisin esterase (FZYM) and it functions by cleaving the ester linkages in FB1 side chains, producing either fully or partially hydrolyzed FB1 and tricarballylic acid(s) (Heinl et al. 2010). The European Food Safety Authority (EFSA) assessed BENT and FZYM and the European Commission approved both of them for use in ruminants, pigs, and poultry to mitigate the harmful effects of AFs and FBs, respectively (EFSA Panel on Additives and Products or Substances used in Animal Feed (FEEDAP), 2016). The BENT and FZYM are marketed by Biomin® GmbH, a division of dsm-firmenich, as Mycofix® Secure and FUMzyme®, respectively. These mycotoxin detoxifiers are frequently tested outside of SSA in experimental conditions that, among other things, do not reflect the majority of SSA’s farming practices. These conditions include temperature, mycotoxin contamination levels, feed composition and management, and vaccination schedules. Furthermore, there has never been a report on the use of BENT and FZYM in feed ingredients contaminated with one or more mycotoxins.

The objective of the present study was to evaluate the safety and efficacy of the mycotoxin detoxifiers FZYM and BENT to reduce the negative effects of AFs and FBs, either separately or in combination, on laying hens, in experimental conditions comparable to those of small-scale commercial farming in the majority of SSA countries. Production performance of the layers was determined by feed intake, egg weight, feed conversion ratio, and egg production, whereas the health of the hens was evaluated by mortality rate, and changes in blood biochemistry and organ weights.

## Material and methods

### Ethical statement

The animals were housed at the International Livestock Research Institute (ILRI), located in Nairobi, Kenya. All methods related to maintaining, euthanizing, and sampling the animals followed the ILRI Animal Care and Use Ethics Committee approval number IACUC-RC2019-03.

### Preparation of experimental AFB1- and FBs-contaminated diets

Maize culture materials containing AFB1 or FBs were obtained according to the methods described by Ochieng et al. (2022). *Aspergillus flavus* and *Fusarium verticillioides* fungal isolates to produce AFs and FBs, respectively, were from the Mycology and Mycotoxin Laboratory, University of Nairobi, Kenya. Using LC-MS/MS methods (Monbaliu et al. 2010), the maize culture materials were examined for major AFs and FBs. Levels up to 88,174 µg AFB1/kg substrate and 1709 µg aflatoxin B2 (AFB2)/kg substrate were measured in maize cultures inoculated with *A. flavus* and levels up to 440,668 µg FB1/kg and 449,056 µg fumonisin B2 (FB2)/kg were found in maize cultures inoculated with *F. verticillioides*.

The control diet (with no additional mycotoxins or detoxifiers) was a commercially supplied basal feed free of growth promoters, antibiotics, and coccidiostats and matched the nutritional requirements for laying hens (Nutrient Requirements of Poultry 1994) as shown in Supplementary Table S1. Using the LC-MS/MS method developed and validated by Sulyok et al. (2006), the control diet’s mycotoxin levels were determined. Supplementary Table S1 shows the concentrations of the major mycotoxins in the diet. All the tested mycotoxins were at trace levels and have been shown in previous studies to be non-toxic to poultry. The diet had AFB1 at a level of 2.26 µg/kg, FB1 at a level of 274.10 µg/kg, and FB2 at a level of 94.98 µg/kg (all below the EU legal or recommended levels in poultry feeds, European Commission 2002, 2006).

The maize culture materials containing AFB1, or FBs, were mixed with 5000 g of the control diet to make a premix, which was then used to formulate the experimental treatment diets contaminated with AFB1 at levels of 54.6 or 546 µg/kg feed and FBs (FB1 + FB2) at a level of 7.9 mg/kg feed. Fumonisin B1 was at a level of 6.08 mg/kg feed whereas FB2 was at a level of 1.80 mg/kg feed in the FBs-contaminated diets. Bentonite and FZYM at doses of 2 g/kg feed and 0.012 g/kg feed, respectively, were added to certain diets. Table 1 shows the 20 dietary treatments.

**Table 1:** The different treatment diets fed to laying hens for 28 days

Treatment N°	AFB1 concentration (µg/kg feed)	FBs concentration (mg/kg feed)	BENT dose (g/kg feed)	FZYM dose (g/kg feed)
T1: Control	/	/	/	/
T2: FBs	/	7.9	/	/
T3: FBs + FZYM	/	7.9	/	0.012
T4: FBs + FZYM + BENT	/	7.9	2	0.012
T5: H AFB1	546	/	/	/
T6: H AFB1 + BENT	546	/	2	/
T7: H AFB1 + BENT + FZYM	546	/	2	0.012
T8: H AFB1 + FBs	546	7.9	/	/
T9: H AFB1 + FBs + BENT	546	7.9	2	/
T10: H AFB1 + FBs + FZYM	546	7.9	/	0.012
T11: H AFB1 + FBs + BENT + FZYM	546	7.9	2	0.012
T12: M AFB1	54.6	/	/	/
T13: M AFB1 + BENT	54.6	/	2	/
T14: M AFB1 + BENT + FZYM	54.6	/	2	0.012
T15: M AFB1 + FBs	54.6	7.9	/	/
T16: M AFB1 + FBs + BENT	54.6	7.9	2	/
T17: M AFB1 + FBs + FZYM	54.6	7.9	/	0.012
T18: M AFB1 + FBs + BENT + FZYM	54.6	7.9	2	0.012
T19: FZYM	/	/	/	0.012
T20: BENT	/	/	2	/

*M AFB1* moderate aflatoxin B1, *H AFB1* high aflatoxin B1, *BENT* bentonite, *FZYM* fumonisin esterase, *FBs* fumonisins

### Experimental birds and housing

Four hundred Isa Brown laying hens, aged 19 weeks (body weight (BW) ± standard deviation = 1.7 ± 0.2 kg), were purchased from a small-scale commercial farm in Kenya. Two weeks were given to the chickens to adapt to their new environment before the feeding trial began. During the adaption period, all the hens were fed the control diet. At the beginning of the 28-day feeding trial, the birds were 21 weeks old, weighed 1.8 ± 0.1 kg, and had laying capacities of above 80%. Twenty birds (four replicates of 5 birds each) were assigned to each of the 20 treatment groups after the birds had their wings banded. Each pen measured approximately 2 m^2^ and was filled with sterilized pine wood shavings on concrete flooring. The pens were naturally lit and had temperatures of between 22 and 25 °C, emulating Kenyan small-scale commercial farming practices. Before placing the hens, the pens were thoroughly cleaned with Hy-Protectol® disinfectant (HighChem, Nairobi, Kenya) and allowed to dry for 3 days. The birds were fed the various treatment diets and water ad libitum for the duration of the 28-day feeding period. The general flock conditions were checked twice a day and in the event that a mortality was recorded, a post-mortem examination was done immediately.

### Production performance; collection of blood, organs, and eggs; and blood biochemistry

#### Production performance parameters

Every day, feed intake (FI) was calculated by deducting the amount of feed left over from the amount of feed that was supplied and corrected for mortalities. Eggs were collected every day, marked with the day they were collected and pen number before being weighed, and stored at 4 °C. Egg production was computed using the number of eggs laid by each hen each day, taking into account the production from all surviving hens. The feed conversion ratio (FCR) was determined as the weight (g) of feed used per weight (g) of egg produced (Zhu et al. 2023). Body weight gain (BWG) was computed by deducting the starting BW from the final BW.

#### Collection of blood, organs, and eggs

At the end of the feeding trial, blood (about 2 mL) was aseptically collected from four birds in each pen via the wing vein using a sterile 23G needle (0.65 mm × 30 mm) and a 2-mL syringe. Blood from two birds/pens was put in 10-mL plain tubes to obtain serum, while the remaining blood from the other two birds was put in sample tubes containing EDTA to obtain plasma. For the latter, after being allowed to stand at room temperature (22–25 °C) for 2 h, each blood sample was centrifuged for 10 min at 4 °C and 3000 rpm. The collected sera and plasma samples were stored at − 20 °C in vials until blood biochemistry analysis and analysis for residues of AFs, respectively. The birds were then weighed and sedated with an intramuscular injection of 3.1 mg/kg BW ketamine hydrochloride (Rotexmedica GmbH, Trittau, Germany) and 0.2 mg/kg BW midazolam (Troikaa, Gujarat, India), followed by euthanasia using an intravenous injection of 86 mg/kg BW pentobarbital (Bayer, Johannesburg, South Africa). Whole liver, spleen, heart, and gizzard were obtained from the same two birds/pens from which plasma samples were taken. The organs were weighed, and the relative organ weight was computed as a proportion of the BW (Saminathan et al. 2018). About 100 g of breast muscle and the entire liver was collected from the same birds from which plasma was obtained and the samples were stored at − 20 °C until they were shipped frozen to be examined for AFs residues. Eggs collected on the final feeding trial day were weighed, shelled, centrifuged, and individually stored in 50-mL Eppendorf tubes at − 20 °C until they were delivered frozen for AFs residue analysis.

#### Blood biochemistry analysis

An automated Cobas C600 biochemical analyzer (Roche Ltd, Horiba-ABX, Montpellier, France) was used to measure the levels of total protein (TP), albumin (ALB), gamma-glutamyl transferase (GGT), and uric acid (UA) in the sera samples according to the manufacturer’s recommended protocols. By deducting the ALB from the TP, the serum globulin (GLB) levels were determined (Sakamoto et al. 2018).

### Analysis of residues of aflatoxins in plasma, liver, muscle tissue, and eggs using UPLC-MS/MS

Using a Moulinette 320 meat grinder (Moulinex, Barcelona, Spain), the liver and breast muscle samples were ground and homogenized. The UPLC-MS/MS methods reported by De Baere et al. (2023) for the determination of AFB1, AFB2, aflatoxin G1 (AFG1), aflatoxin G2 (AFG2), AFM1, and aflatoxin M2 (AFM2) in plasma, muscle, liver, and eggs were used. The methods were in-house validated for freeze–thaw stability, matrix effect, linearity, precision, accuracy, specificity, limit of detection (LOD), limit of quantification (LOQ), and extraction recovery (RE). Egg, muscle, liver, and plasma samples from healthy, untreated chickens were used to prepare matrix-matched blank and spiked samples for method validation. Details of the method validation are provided by De Baere et al. (2023). The LOQ of AFB1, AFB2, AFG1, AFG2, and AFM1 in plasma was 0.05 ng/mL, while the LOQ of AFM2 in the same matrix was 0.10 ng/mL. In chicken liver, the LOQ was 0.05 µg/kg for AFB1 and 0.10 µg/kg for AFB2 and AFM1, whereas it was 0.25 µg/kg for AFG1 and AFG2 and 0.5 µg/kg for AFM2. For chicken muscle, the LOQ for AFB1 was 0.05 µg/kg, 0.10 µg/kg for AFM1, and 0.25 µg/kg for AFB2, AFG1, and AFG2. In chicken egg, the LOQ for AFB1, AFB2, and AFM1 was 0.025 µg/kg, while it was 0.050 µg/kg for AFG1 and AFG2 and 0.50 µg/kg for AFM2. For chicken plasma, the calculated LOD values were from 0.0029 to 0.0300 ng/mL; for chicken liver and muscle, the LODs were between 0.006 and 0.040 µg/kg and between 0.002 and 0.097 µg/kg for egg.

### Statistical analysis and carry-over factors

R (R Core Team 2020) was used to analyze all the data, which are presented as least squares means and standard error of the mean. Prior to analysis, non-linear data according to the Kolmogorov–Smirnov test were first square root converted. While individual birds were employed for other analyses, the pen served as the experimental unit for the FI analysis. Linear mixed effects modelling from the R package was used with the pen serving as the random variable (Tsiouris et al. 2021). The means of the various treatment groups were compared using predefined contrast analysis (Chowdhury et al. 2005). The threshold for statistical significance in the Tukey post hoc analysis was set at *p* < 0.05.

Aflatoxin residues in plasma, muscle, liver, and eggs were deemed positive if their concentration was higher than the limit of detection (LOD), and half of the LOQ value was applied to samples that were above LOD but below LOQ (Kemboi et al. 2023).

The carry-over factors of AFs from feed into plasma, liver, muscle, and egg were expressed as the ratio of the mycotoxin concentration (µg/kg) in each tissue relative to the mycotoxin concentration (µg/kg) in feed × 100.

## Results

### Production performance

In groups fed the diet with high AFB1 alone (T5) and the diet with both high AFB1 and FBs and supplemented with FZYM (T10), two mortalities were observed that were unrelated to the treatment diets as indicated in the post-mortem reports (not shown). Table 2 shows the average daily FI, FCR, egg weight, and egg production during the 28-day feeding trial. Compared to the control diet (T1), the production of eggs dropped by 5% (*p* = 0.0302) in birds fed high AFB1 alone (T5). When BENT and FZYM were added to the diets with high AFB1 (T7), egg production increased by 7% (*p* = 0.0066) compared to diets with high AFB1 without the detoxifiers (T5). Dietary AFB1, FBs, or both, did not significantly alter egg weights (*p* > 0.05). However, compared to a diet contaminated with moderate AFB1 and FBs and no detoxifiers (T15), egg weight increased by 3% (*p* = 0.0416) when both FZYM and BENT were supplemented into the moderate AFB1 and FBs diet (T18). The laying hens’ FCR and FI were unaffected by the various treatments. Moreover, neither BW nor BWG was changed by the treatments (results not shown).

**Table 2:** Average daily feed intake, egg weight, feed conversion ratio, and egg production of laying hens fed different diets. Each treatment diet included 20 birds

Treatment N°	Feed intake (g/bird per day)	Egg weight (g/egg)	Feed conversion ratio (g of feed/g of egg)	Egg production (%)
T1: Control	133.7	60.0^ac^	2.23	92.9^bcd^
T2: FBs	133.6	59.5^ab^	2.24	91.4^ad^
T3: FBs + FZYM	133.6	60.8^ac^	2.20	93.2^ cd^
T4: FBs + FZYM + BENT	133.6	60.0^ac^	2.23	92.9^bcd^
T5: H AFB1	133.4	59.7^ac^	2.24	88.2^a^
T6: H AFB1 + BENT	133.8	60.9^bc^	2.20	91.8^ad^
T7: H AFB1 + BENT + FZYM	133.4	59.4^a^	2.25	94.1^d^
T8: H AFB1 + FBs	133.5	59.8^ac^	2.24	90.2^ad^
T9: H AFB1 + FBs + BENT	133.4	60.2^ac^	2.22	89.1^ac^
T10: H AFB1 + FBs + FZYM	133.6	61.0^c^	2.21	91.6^ad^
T11: H AFB1 + FBs + BENT + FZYM	133.4	60.1^ac^	2.22	89.1^ac^
T12: M AFB1	133.7	59.8^ac^	2.24	91.8^ad^
T13: M AFB1 + BENT	133.6	60.3^ac^	2.22	90.7^ad^
T14: M AFB1 + BENT + FZYM	133.6	60.3^ac^	2.22	88.9^ab^
T15: M AFB1 + FBs	133.8	59.4^a^	2.26	89.6^ac^
T16: M AFB1 + FBs + BENT	133.8	60.2^ac^	2.23	92.5^bcd^
T17: M AFB1 + FBs + FZYM	133.4	59.8^ac^	2.24	91.6^ad^
T18: M AFB1 + FBs + BENT + FZYM	133.7	60.9^bc^	2.20	93.2^ cd^
T19: FZYM	133.6	60.3^ac^	2.22	92.5^bcd^
T20: BENT	133.6	60.0^ac^	2.23	92.1^ad^
SEM	0.2	0.5	0.02	1.48

Data are presented as least square means (LSM) and standard error of the mean (SEM) for 20 birds per treatment. Values within the same column not sharing a common superscript differ significantly (*p* < 0.05) following a Tukey post hoc test. The feed conversion ratio was calculated by dividing the sum of feed consumed per hen per day by the weight of the egg produced. *FBs* fumonisins, *H AFB1* high aflatoxin B1, *M AFB1* moderate aflatoxin B1, *FZYM* fumonisin esterase, *BENT* bentonite

### Relative weight of organs

Table 3 shows the relative organ weights of the layers from the various treatments as a proportion of BW. In comparison to the control diet (T1), the relative liver weight of the layers increased considerably due to diets with high AFB1 only (T5) or high AFB1 and FBs (T8) by 9% (*p* = 0.0148) and 8% (*p* = 0.0378), respectively. Additionally, compared to the control diet (T1), the diet with moderate AFB1 and FBs (T15) increased the layers’ relative liver weight by 8% (*p* = 0.0323). The relative liver weights were decreased when BENT was added to the AFB1-contaminated diets, although the decreases were not statistically significant (*p* > 0.05). In layers fed high AFB1 alone diet (T5), the relative spleen weight increased by 14% (*p* = 0.0158) and 12% (*p* = 0.0386) as opposed to the diets containing both AFB1 and FBs (T8) and the control diet (T1), respectively. When compared to a diet with high AFB1 alone and no detoxifiers (T5), adding both BENT and FZYM to the diet with high AFB1 (T7) significantly decreased the relative spleen weights by 16% (*p* = 0.0289). The laying hens fed high AFB1 alone (T5) or both moderate AFB1 and FBs (T15) had considerably greater relative gizzard weights by 10% (*p* = 0.0141) and 13% (*p* = 0.0356), respectively, than those fed the control diet (T1). Furthermore, hens fed diets containing both moderate AFB1 and FBs (T15) had relative gizzard and heart weights that were 9% (*p* = 0.0269) and 10% (*p* = 0.0205) greater, respectively, than chickens fed diets containing only moderate AFB1 (T12). When BENT was added to a diet with moderate levels of AFB1 and FBs (T16) or FZYM was added to the same diet (T17), the relative weights of the heart and gizzard were reduced (*p* < 0.05) compared to the contaminated diet without the detoxifiers (T15). None of the organs under investigation was affected by diets containing only FZYM (T19) or BENT (T20) (*p* > 0.05).

**Table 3:** Relative weights of liver, spleen, gizzard, and heart (% body weight) and blood biochemical parameters of the laying hens at the end of the feeding trial (28 days)

Treatment N°	Relative liver weight (%)	Relative spleen weight (%)	Relative gizzard weight (%)	Relative heart weight (%)	Total protein (g/L)	Albumin (g/L)	Globulin (g/L)	Uric acid (mg/dL)
T1: Control	1.47^ab^	0.37^acd^	1.25^a^	0.63^ad^	6.22^ab^	4.06^ac^	4.71^a^	1.48^a^
T2: FBs	1.52^ad^	0.34^a^	1.29^abc^	0.58^a^	5.85^a^	3.62^a^	4.58^a^	1.54^ab^
T3: FBs + FZYM	1.44^a^	0.35^ac^	1.26^ab^	0.62^abc^	6.64^ac^	4.29^bc^	5.06^ac^	2.18^ cd^
T4: FBs + FZYM + BENT	1.54^ad^	0.38^ae^	1.24^a^	0.62^abc^	7.00^bc^	4.55^c^	5.32^ac^	1.99^ cd^
T5: H AFB1	1.60^d^	0.42^e^	1.38^ cd^	0.64^bd^	7.02^bc^	4.49^bc^	5.40^ac^	2.00^ cd^
T6: H AFB1 + BENT	1.56^bcd^	0.38^ae^	1.29^abc^	0.63^ad^	7.21^c^	4.59^c^	5.95^c^	1.80^c^
T7: H AFB1 + BENT + FZYM	1.57^bcd^	0.36^acd^	1.36^bd^	0.65^ cd^	6.91^bc^	4.47^bc^	5.26^ac^	2.16^ cd^
T8: H AFB1 + FBs	1.58^ cd^	0.36^acd^	1.33^ad^	0.64^bd^	6.66^ac^	4.35^bc^	5.03^ac^	1.97^d^
T9: H AFB1 + FBs + BENT	1.58^ cd^	0.36^acd^	1.30^abc^	0.63^ad^	7.09^bc^	4.56^c^	5.42^ac^	2.01^bcd^
T10: H AFB1 + FBs + FZYM	1.57^bcd^	0.39^bce^	1.28^abc^	0.63^ad^	6.73^bc^	4.33^bc^	5.16^ac^	1.87^ac^
T11: H AFB1 + FBs + BENT + FZYM	1.54^ad^	0.37^e^	1.31^ad^	0.63^ad^	6.57^ac^	4.18^bc^	5.07^ac^	2.12^ cd^
T12: M AFB1	1.57^bcd^	0.36^acd^	1.29^abc^	0.61^abc^	6.44^ac^	4.22^bc^	4.87^ab^	2.09^ cd^
T13: M AFB1 + BENT	1.48^ac^	0.36^acd^	1.29^abc^	0.62^ad^	6.92^bc^	4.44^bc^	5.30^ac^	1.95^ac^
T14: M AFB1 + BENT + FZYM	1.54^ad^	0.36^acd^	1.34^ad^	0.61^abc^	6.80^bc^	4.31^bc^	5.25^ac^	2.24^ cd^
T15: M AFB1 + FBs	1.59^ cd^	0.37^acd^	1.41^d^	0.67^d^	6.80^bc^	4.10^ac^	5.41^ac^	1.93^ac^
T16: M AFB1 + FBs + BENT	1.51^ad^	0.34^ac^	1.30^abc^	0.60^abc^	6.76^bc^	4.23^bc^	5.27^ac^	2.23^ cd^
T17: M AFB1 + FBs + FZYM	1.52^ad^	0.37^acd^	1.30^abc^	0.61^abc^	6.74^bc^	4.07^ac^	5.37^ac^	1.85^ac^
T18: M AFB1 + FBs + BENT + FZYM	1.53^ad^	0.34^ab^	1.31^ad^	0.60^abc^	7.12^c^	4.31^bc^	5.66^bc^	2.45^d^
T19: FZYM	1.50^ac^	0.40^de^	1.29^abc^	0.63^ad^	6.70^ac^	3.93^ab^	5.71^bc^	2.10^ cd^
T20: BENT	1.51^ad^	0.39^ce^	1.31^ad^	0.59^ab^	6.38^ac^	4.04^ac^	4.94^ab^	2.04^ cd^
SEM	0.04	0.02	0.04	0.02	0.32	0.20	0.32	0.18

Data are presented as least square means (LSM) and standard error of the mean (SEM) for 8 birds per treatment. Values within the same column not sharing a common superscript differ significantly (*p* < 0.05) according to a Tukey post hoc test. *FBs* fumonisins, *H AFB1* high aflatoxin B1, *M AFB1* moderate aflatoxin B1, *FZYM* fumonisin esterase, *BENT* bentonite

### Biochemical parameters

Table 3 also shows the alterations in serum TP, ALB, GLB, and UA brought on by the various experimental diets. When the diet contaminated with FBs was supplemented with both FZYM and BENT (T4), the concentrations of TP and ALB were considerably increased by 20% (*p* = 0.0289) and 26% (*p* = 0.0011), respectively, compared to the diet with FBs only and no detoxifiers (T2). Additionally, compared to a diet with FBs only (T2), the addition of FZYM to a diet with FBs only (T3) raised the serum ALB by 19% (*p* = 0.0305). When BENT was added to a high AFB1 only diet (T6), serum TP and GLB levels were elevated by 16% (*p* = 0.0297) and 26% (*p* = 0.0069), respectively, in comparison to the control diet (T1). When compared to the control diet (T1), the addition of FZYM and BENT to a diet with moderate AFB1 and FBs (T18) increased the GLB by 21% (*p* = 0.0289), while the diet with FZYM alone (T19) increased the serum GLB by 20% (*p* = 0.0372). The various treatments significantly affected serum UA concentrations. Serum UA concentrations in the layers fed diets with high AFB1 only (T5), high AFB1 and FBs (T8), or moderate AFB1 (T12) were higher than those on the control diet (T1). In comparison to the control diet (T1), the addition of FZYM, BENT, or both to contaminated diets (T3, T4, T6, T7, T9, T11, T14, T16, and T18) also elevated the UA concentrations of the layers (*p* < 0.05). The serum UA of layers fed diets containing FZYM alone (T19) or BENT alone (T20) were greater by 42% (*p* = 0.0111) and 38% (*p* = 0.0238), respectively, than those fed the control diet (T1). The various treatments had no effect on serum GGT (results not shown).

### Aflatoxins residues in plasma, liver, muscle, and egg

Aflatoxin B1 residues were found in plasma, liver, and egg samples (Table 4). In breast muscle samples from all experimental groups, all of the tested AFs were below detectable levels (results not shown). Levels of AFB1 were in the range of LOQ to 0.639 µg/kg in liver, LOQ to 0.063 ng/mL in plasma, and LOQ to 0.040 µg/kg in eggs. The livers of layers fed both high AFB1 and FBs (T8) had the highest level of AFB1 residues (0.64 µg/kg), but this value did not differ statistically from AFB1 residues found in the livers of layers fed a diet with high AFB1 only (T5) (*p* > 0.05). Layers fed moderate AFB1 alone (T12) or with FBs (T15) had levels of liver AFB1 residues of 0.22 µg/kg and 0.10 µg/kg, respectively, and these values did not differ statistically from each other. When compared to a diet with high AFB1 (T5), the addition of both BENT and FZYM to the diet (T7) significantly reduced the AFB1 residues in the liver by 82% (*p* = 0.0044). Similarly, the liver samples from birds fed high AFB1 and FBs and supplemented with BENT (T9) or FZYM (T10) showed a significant reduction in AFB1 residues of 71% and 81%, respectively, when compared to the same diet without the detoxifiers (T8) (*p* < 0.001).

**Table 4:** Aflatoxin B1 (AFB1) concentrations in laying hens’ plasma (ng/mL), liver, and eggs (µg/kg) from the different treatments at the end of the feeding period (28 days)

Treatment N^o^	AFB1 concentration (ng/mL or µg/kg)		
	Plasma	Liver	Eggs
T1: Control	< LOQ^a^	ND^a^	ND^a^
T2: FBs	< LOQ^a^	< LOQ^a^	ND^a^
T3: FBs + FZYM	< LOQ^a^	ND^a^	ND^a^
T4: FBs + FZYM + BENT	< LOQ^a^	ND^a^	ND^a^
T5: H AFB1	0.063^b^	0.439^cde^	0.040^ g^
T6: H AFB1 + BENT	0.059^b^	0.327^bd^	< LOQ^de^
T7: H AFB1 + BENT + FZYM	< LOQ^a^	0.080^ab^	< LOQ^c^
T8: H AFB1 + FBs	< LOQ^a^	0.639^e^	0.028^f^
T9: H AFB1 + FBs + BENT	< LOQ^a^	0.187^abc^	0.025^ef^
T10: H AFB1 + FBs + FZYM	< LOQ^a^	0.124^ab^	< LOQ^cd^
T11: H AFB1 + FBs + BENT + FZYM	< LOQ^a^	0.457^de^	< LOQ^e^
T12: M AFB1	ND^a^	0.215^ad^	< LOQ^ab^
T13: M AFB1 + BENT	ND^a^	< LOQ^a^	ND^a^
T14: M AFB1 + BENT + FZYM	< LOQ^a^	< LOQ^a^	< LOQ^ab^
T15: M AFB1 + FBs	0.025^a^	0. 101^ab^	< LOQ^ab^
T16: M AFB1 + FBs + BENT	ND^a^	< LOQ^a^	< LOQ^bc^
T17: M AFB1 + FBs + FZYM	< LOQ^a^	< LOQ^a^	< LOQ^ab^
T18: M AFB1 + FBs + BENT + FZYM	ND^a^	ND^a^	< LOQ^ab^
T19: FZYM	ND^a^	< LOQ^a^	ND^a^
T20: BENT	ND^a^	ND^a^	ND^a^
SEM	0.012	0.105	0.002

*LOQ* limit of quantification (0.050 µg/kg or ng/mL for AFB1 residues in liver and plasma samples and 0.025 µg/kg for AFB1 residues in egg samples). *ND* not detected. Data are presented as least square means (LSM) and standard error of the mean (SEM) for 8 birds per treatment. *FBs* fumonisins, *H AFB1* high aflatoxin B1, *M AFB1* moderate aflatoxin B1, *FZYM* fumonisin esterase, *BENT* bentonite

Aflatoxin M1 was present in plasma and liver of birds given high or moderate AFB1 alone or with FBs or the detoxifiers, although below the LOQ of 0.050 ng/mL or 0.10 µg/kg, respectively. Trace levels (below LOQs) of AFG1, AFG2, AFB2, and AFM2 were found in the liver and plasma samples of birds that were given high AFB1-contaminated diets (results not shown).

Table 4 shows that AFB1 was only found in eggs above the LOQ of 0.025 µg/kg in birds fed diets containing high AFB1 alone (T5), or in combination with FBs (T8), or in diets containing both high AFB1, FBs, and supplemented with BENT (T9). When comparing the transfer of AFB1 from feeds into eggs between layers fed a diet with high AFB1 only (T5) and a diet with both high AFB1 and FBs (T8), the results showed a significant difference (*p* < 0.001). The addition of BENT (T6) or both BENT and FZYM (T7) significantly reduced the carry-over of AFB1 into the eggs when compared to a diet with high AFB1 without the detoxifiers (T5) (*p* < 0.001). Similarly, supplementing FZYM (T10) or both FZYM and BENT (T11) into a diet containing high AFB1 and FBs (T8) statistically reduced the concentration of AFB1 transferred into eggs (*p* < 0.0010 and *p* = 0.0183, respectively).

Eggs from layers fed diets high in AFB1 (T5-T11) showed detectable levels of AFM1, albeit below the LOQ of 0.025 µg/kg. Other tested AFs including AFB2, AFM2, AFG1, and AFG2 were not found in eggs from all treatment groups (data not shown).

Table 5 shows the AFB1 carry-over factors from feed into plasma, liver, and eggs. Birds fed diets containing high levels of AFB1 and FBs (T8) had liver samples with the greatest carry-over factor (0.12%) overall. When comparing AFB1 carry-over factors from feed to eggs with carry-over factors to liver and plasma from the same laying hens, the former showed lower carry-over factors.

**Table 5:** Carry-over factors (%) of AFB1 from feed to plasma, liver, and eggs of laying hens fed diets contaminated with the high AFB1 level (546 µg/kg feed), alone or in combination with FBs, or BENT, and/or FZYM for 28 days

Treatment N°	Carry-over factors (%)		
	Plasma	Liver	Eggs
T5: H AFB1	0.012	0.080	0.007
T6: H AFB1 + BENT	0.010	0.060	NA
T7: H AFB1 + BENT + FZYM	NA	0.015	NA
T8: H AFB1 + FBs	NA	0.117	0.005
T9: H AFB1 + FBs + BENT	NA	0.034	0.005
T10: H AFB1 + FBs + FZYM	NA	0.023	NA
T11: H AFB1 + FBs + BENT + FZYM	NA	0.084	NA

Carry-over factors (%) from feed into plasma, liver, and eggs expressed as a percentage of the concentration of AFB1 in tissues (µg/kg) compared to the concentration of AFB1 in feed (µg/kg). *NA* not applicable, *FBs* fumonisins, *H AFB1* high aflatoxin B1, *M AFB1* moderate aflatoxin B1, *FZYM* fumonisin esterase, *BENT* bentonite

## Discussion

The results of this study demonstrated that some of the detrimental effects of FBs and AFB1 were mitigated by FZYM and BENT, respectively. Overall, the different treatments had no effect on FI and FCR. Zaghini et al. (2005) also found no changes in the FI of laying hens fed diets with AFB1 up to levels of 2500 µg/kg feed for 4 weeks. However, when AFB1 and DON (both at 2000 µg/kg feed) were fed to commercial-strain hens during their peak production, reduced FI and high FCR were observed (Lee et al. 2012). In the latter study, usage of higher concentrations of AFB1 might explain the observed differences.

The highest dosage of AFB1 used in the present study was shown to lower egg production. Fernandez et al. (1994) also observed a reduction in egg production in laying hens fed AFB1 at a concentration of 5000 µg/kg feed for 32 days. However, when AFB1 was fed to laying hens at doses nearly similar to the current study (500 µg/kg) for 8 weeks, no effect on egg production was noted (Oliveira et al. 2000). The differences in the breed sensitivity of the Babcock hens in the latter study versus Isa Brown in the current study could be the cause of the discrepancy in the results. In the current study, the addition of both FZYM and BENT to diets containing high or moderate AFB1 and FBs improved the egg production and egg weights, respectively. These results suggest the need for multi-component detoxifiers since poultry diets are often contaminated with multiple mycotoxins.

The liver is the main target organ for AFs and FBs toxicities and an increase in the relative weight of the livers from layers fed AFB1 alone or in combination with FBs was expected. Laying hens fed dietary AFB1 at levels of 150–5000 µg/kg feed showed increased liver weights as a result of lipid accumulation (Fernandez et al. 1994; Zhao et al. 2021). According to Lee et al. (2012), laying hens fed dietary AFB1 and DON, both at levels of 1500 or 2000 µg/kg feed, also had increased liver weights. The increased liver weights in the latter study were associated with AFB1. The present study showed that lower liver weights were observed when BENT was added to the AFB1-contaminated diets; however, these differences were not statistically significant, and this could be because the diets contained high levels of AFB1. Shannon et al. (2017) also reported that BENT could not fully prevent the increase in liver weights in broiler hens fed a very high dietary AFB1 dose (2000 µg/kg) from day 1 to day 21. Thus, there is a need to determine the dosage of a detoxifier based on the level of mycotoxin in a feed. In the present study, laying hens given high AFB1 alone showed increased spleen weights. On the other hand, Zhao et al. (2021) found that layers fed AFB1 (150 µg/kg feed) along with DON (1500 µg/kg feed) and OTA (120 µg/kg feed) had reduced spleen weights. The additional negative effects of DON and OTA in the latter study may be the cause of the observed discrepancies in the results. The spleen is an organ of the immune system, and any impairments therein suggest interferences with the immune system. The laying hens’ gizzard weights in the current study increased when they were fed diets contaminated with AFB1. However, in studies conducted with broiler chickens, feeding AFB1 in the range of 20 to 500 µg/kg for 35 days had no effect on the weight of the gizzard (Mesgar et al. 2022; Ochieng et al. 2023; Saminathan et al. 2018). When compared to the effects of AFB1-contaminated diets alone, the combination of AFB1 and FBs caused more severe effects on the weights of the spleen, gizzard, and heart, suggesting interactions between the two mycotoxins. A study by Huff et al. (1988) revealed that interactions between mycotoxins can result in enhanced harmful effects on the health and production of chickens. Pappas et al. (2016) observed that broiler hens fed diets containing 100 µg/kg feed of both OTA and AFB1 for 42 days showed increased heart weight.

In the present study, the detrimental effects of the mycotoxins on the weights of the chickens’ hearts and spleens were reduced when BENT, FZYM, or both were added to the contaminated diets. This result supports the reports on the efficacy of BENT and FZYM for usage in poultry to reduce effects of AFB1 and FBs as previously documented in other studies (EFSA Panel on Additives and Products or Substances used in Animal Feed (FEEDAP), 2020; Shannon et al. 2017). The FZYM only at levels of 0.012 g/kg feed or the BENT only at levels of 2 g/kg feed were safe and had no effect on any of the organs examined, indicating the safety of these detoxifiers.

Serum TP, ALB, and GLB concentrations were higher in birds given contaminated diets but supplemented with FZYM, BENT, or both, showing the mycotoxin mitigation effects of the detoxifiers. Grenier et al. (2017)) and Shannon et al. (2017) also observed that FZYM and BENT reduced the effects of FBs and AFB1, respectively, on the blood biochemistry of broiler chickens. The present study showed an increase in serum UA concentrations of hens fed AFB1-contaminated diets or contaminated diets supplemented with FZYM, BENT, or both. In contrast, Swamy et al. (2002) reported that broiler hens exposed to AFs have lower serum UA levels which could indicate changes in renal filtration and reabsorption rates. Studies with broiler chickens revealed no changes in the serum UA levels due to dietary AFB1 at dosages of 100 to 220 µg/kg feed (Ochieng et al. 2023; Oğuz et al. 2002). The various treatment diets in the current study had no effect on the serum GGT concentration. However, Fernandez et al. (1994) observed elevated serum GGT levels in laying hens fed AFB1 at concentrations nearly five times greater (2500 µg/kg feed) than the present study. An increase in blood enzymes, such as GGT, can signify damage of hepatocytes and can be used to diagnose for mycotoxin exposure long before significant clinical symptoms manifest (Tessari et al. 2010).

In the present study, AFB1 was found above LOQ in plasma, liver, and egg samples from layers fed AFB1-contaminated diets. Breast muscle samples from all experimental groups did not contain any of the AFs that were tested. The maximum AFB1 residual concentration of 0.64 µg/kg corresponding to a carry-over factor of 0.12% was found in liver samples of layers fed a diet with both FBs and AFB1. The present study’s carry-over was slightly lower than in another study with laying hens where diets contaminated with AFB1 at levels of 894 µg/kg feed resulted in liver AFB1 residues of 1.59 µg/kg and thus a calculated carry-over factor of 0.18% (Herzallah 2013). Other researchers found that feeding 2500 µg/kg feed of AFB1 to laying hens produced up to 4.13 µg/kg of liver AFB1 residues in one trial and 2.21 µg/kg in another, and corresponding calculated carry-over factors of 0.17% and 0.09%, respectively (Rizzi et al. 2003; Zaghini et al. 2005). For a short-term 7-day feeding trial, Trucksess et al. (1983) found that laying hens fed diets with extremely high AFB1 levels of 8000 µg/kg feed had a calculated lower carry-over factor of 0.01% when compared to the present study. In our previous work, the highest AFB1 residue level of 0.12 µg/kg corresponding to a calculated carry-over factor of 0.06% was found in liver samples of broiler chickens fed dietary AFB1 at concentrations of 220 µg/kg feed and 17,430 µg FB1 + FB2/kg feed (Ochieng et al. 2023). The differences in the mycotoxin concentrations in feeds, the length of exposure time, and the sensitivity of the breed of hens used in the trials can contribute to the discrepancies in the carry-over factors seen in the various research works. Residues of AFB1 of up to 16.36 µg/kg were found in field surveys of chicken liver samples taken from markets and slaughterhouses, suggesting that the hens were exposed to AFB1, particularly through contaminated diets (Amirkhizi et al. 2015; Iqbal et al. 2014; Sineque et al. 2017).

In comparison to plasma, eggs, and muscle samples, the current study’s findings showed that liver samples had the highest levels of AFB1, and these findings are consistent with those of previous studies (Bintvihok & Kositcharoenkul 2006; Trucksess et al. 1983). Aflatoxin B1 is metabolized in the liver into AFB1-8,9-epoxide, which can then bind to DNA, RNA, or other macromolecules, including proteins in the liver. Additionally, the epoxide can cause malignant growths by deactivating antioxidant enzymes (Yunus et al. 2011).

The BENT utilized in this study decreased the concentration of AFB1 that accumulated in the liver of layers fed diets contaminated with AFB1, supporting the findings of Bhatti et al. (2018) that BENT can bind to AFB1 and the binder-AFB1 complex is excreted through feces, thereby reducing the level of AFB1 that bioaccumulates in organs.

Both plasma and liver samples of layers fed diets containing high levels of AFB1 or a diet consisting only of BENT showed traces of AFM1 (below the LOQ). One of the hydroxylated metabolites of AFB1 is AFM1 and it is frequently found in the tissues, milk, or eggs of animals that have been exposed to AFB1 (Kemboi et al. 2023). Trucksess et al. (1983) reported that AFM1 in the concentrations ranges of 0.04 to 0.10 µg/kg was present in kidney samples from laying hens fed AFB1 at extremely high concentrations of 8000 µg/kg feed for a short period of 7 days.

Other AFs examined in this study such as AFB2, AFG1, AFG2, and AFM2 were also found (below the LOQ values) in plasma and liver samples of the layers that were fed diets contaminated with AFB1. These AFs make up a very minor portion of all naturally produced AFs and are rarely found in chicken tissues (Bintvihok & Kositcharoenkul 2006; Okoth et al. 2018).

In the present study, only egg samples taken from layers fed diets with the highest dosage of AFB1 contained residues of AFB1. The highest concentration of AFB1 residues of 0.040 µg/kg (carry-over factor of 0.007%) found in layers fed AFB1 only was significantly higher than the concentration of 0.028 µg/kg found in the egg samples of layers fed both AFB1 and FBs. Future research should look into the effects of mycotoxins' interactions on the transmission of AFB1 into eggs, as this study showed. The majority of research works have assessed the transmission of mycotoxins from feed to eggs in the presence of a single mycotoxin contamination. Oliveira et al. (2000) reported that eggs from laying hens that consumed 500 µg/kg of dietary AFB1 had AFB1 residues of 0.16 µg/kg, which translates to a computed carry-over factor of 0.032%. There may have been a greater accumulation and transfer of AFB1 from feeds into eggs in the latter trial since the feeding time was double (8 weeks) compared to the present study (4 weeks). According to Trucksess et al. (1983), residues of AFB1 of 0.2 µg/kg resulting in a calculated carry-over factor of 0.04% were observed in egg samples of laying hens fed very high concentrations of AFB1 (8000 µg/kg feed) for just 7 days. The latter study’s shorter feeding duration combined with likely low analysis accuracy at low AFB1 levels may have led to the low carry-over observed. In field surveys conducted in SSA, levels of AFB1 residues of up to 7.6 µg/kg were found in egg samples from farms and markets (Tatfo Keutchatang et al. 2022; Tchana et al. 2010). Research done outside SSA showed that egg samples taken from markets and slaughterhouses had AFB1 levels ranging from 0.3 to 5.8 µg/kg (Herzallah 2009; Iqbal et al. 2014). Wang et al. (2018) found up to 168 µg/kg of AFB1 residues in a single egg sample taken from a market.

In the present study, AFB1 carry-over factor into eggs was decreased when BENT was added to diets contaminated with AFB1, suggesting that BENT could bind to AFB1 and decrease its gastrointestinal absorption and subsequent transfer into eggs.

In conclusion, this study demonstrated that feeding laying hens either high (546 µg/kg) or moderate (54.6 µg/kg) AFB1 or FBs (7.9 mg/kg) alone or in combination had no influence on the hens’ FCR and FI. On the other hand, the contaminated diets decreased egg production, increased gizzard, liver, and spleen weights, and elevated serum uric acid levels. When compared to the effects of a single mycotoxin, the interactions between AFB1 and FBs had a significant effect on the weights of the spleen, heart, and gizzard as well as the concentration of AFB1 residues in eggs. Residues of AFB1 (maximum 0.64 µg/kg) and trace levels of AFM1 (below LOQ values) were found in plasma, liver, and egg samples of laying hens that were fed AFB1-contaminated diets. The addition of BENT and/or FZYM to contaminated diets reduced the individual or combined effects of AFB1 and FBs on changes in blood biochemistry, organ weights, egg production, and egg weight. The detoxifiers also decreased the level of AFB1 that accumulated in the liver and eggs. Therefore, the mycotoxin detoxifiers provided an appropriate way to mitigate the detrimental effects of AFB1 and FBs on the productivity and health of laying hens as well as decreased the concentration of AFB1 that was transferred into chicken products, guaranteeing the safety of these food items, especially in SSA where mycotoxin monitoring along the food chain is not consistently conducted.

## Supplementary Information

Below is the link to the electronic supplementary material.

Supplementary file1 (DOCX 25.9 KB)

## Data Availability

No datasets were generated or analyzed during the current study.
