# Supplementary material for: Aflatoxins and fumonisins co-contamination effects on laying hens and use of mycotoxin detoxifiers as a mitigation strategy
Source: Mycotoxin Res. 2024 Oct 15;41(1):63–75. doi: 10.1007/s12550-024-00566-x (PMC11759475; doi:10.1007/s12550-024-00566-x)
Supplement: Supplementary file 1 — Supplementary file1 (DOCX 25.9 KB) [file 12550_2024_566_MOESM1_ESM.docx]

**Supplementary material: aflatoxins and fumonisins co-contamination effects on laying hens and use of mycotoxin detoxifiers as a mitigation strategy**

Phillis E. Ochieng ^a, b^, David C. Kemboi ^b, c^, Sheila Okoth ^d^, Siegrid De Baere ^b^, Etienne Cavalier ^e^, Erastus Kang’ethe ^f^, Barbara Doupovec ^g^, James Gathumbi ^h^, Marie-Louise Scippo ^a^, Gunther Antonissen ^b, i^, Johanna F. Lindahl ^j, k, l, *^, and Siska Croubels ^b, *^

^a^ Department of Food Sciences, Laboratory of Food Analysis, Faculty of Veterinary Medicine, University of Liège, Liège 4000, Belgium

^b^ Department of Pathobiology, Pharmacology and Zoological Medicine, Laboratory of Pharmacology and Toxicology, Faculty of Veterinary Medicine, Ghent University, Merelbeke 9820, Belgium

^c^ Department of Animal Science, Chuka University, P.O. Box 109-60400, 00625, Chuka, Kenya

^d^ Department of Biology, Faculty of Science and Technology, University of Nairobi, P.O. Box 30197-00100, Nairobi, Kenya

^e^ Department of Clinical Chemistry, Center for Interdisciplinary Research on Medicines (CIRM), University of Liège, University Hospital of Liège, Liège 4000, Belgium

^f^ Consultant, 00100, Nairobi, Kenya

^g^ dsm-firmenich Animal Nutrition and Health R&D Center Tulln, 3430 Tulln, Austria

^h^ Department of Veterinary Pathology, Microbiology, and Parasitology, Faculty of Veterinary Medicine, University of Nairobi, P.O. Box 29053-00100, Nairobi, Kenya

^i^ Chair Poultry Health Sciences, Faculty of Veterinary Medicine, Ghent University, Merelbeke 9820, Belgium

^j^ International Livestock Research Institute (ILRI), P.O. Box 30709-00100, Nairobi, Kenya

^k^ Department of Medical Biochemistry and Microbiology, Uppsala University, Uppsala SE-751 05, Sweden

^l^ Department of Clinical Sciences, Swedish University of Agricultural Sciences, Uppsala SE-750 07, Sweden

^*^Corresponding authors

*Email address*: [Siska.Croubels@ugent.be](mailto:Siska.Croubels@ugent.be) (S. Croubels) and [johanna.lindahl@imbim.uu.se](mailto:johanna.lindahl@imbim.uu.se) (J.F. Johanna)

**Table S1**: Chemical and mycotoxin composition of control diet and the EU regulatory/guidance values for the major mycotoxins

| **Layer Feed** | |  |
| --- | --- | --- |
| **Ingredient** | **(%)** |  |
| Dry Matter | 92.05 |  |
| Ash | 15.61 |  |
| Ether Extract (crude lipids) | 3.44 |  |
| Crude protein | 15.20 |  |
| Crude fibre | 6.88 |  |
| Nitrogen Free Extract (Soluble carbohydrates) | 58.89 |  |
| Calcium | 1.12 |  |
| Phosphorus | 0.49 |  |
| Potassium | 0.94 |  |
| **Mycotoxin** | **concentration (µg/kg)** | **^1^EU regulatory/guidance value (µg/kg)** |
| Aflatoxin B1 | 2.26 | 20 |
| Aflatoxin B2 | ND | - |
| Aflatoxin G1 | 0.53 | - |
| Fumonisin B1 | 274.10 | 20,000 (Fumonisin B1+B2) |
| Fumonisin B2 | 94.98 |  |
| Deoxynivalenol | 804.82 | 5,000 |
| Zearalenone | 1,147.48 | 250 |
| Ochratoxin A | ND | 100 |
| T-2 toxin | ND | 250 |

ND; Not detected, -; Not available, ^1^EU regulatory/guidance value according to European Commission (European Commission., 2002, 2006b, 2006a)

**References**

European Commission. (2002). Directive 2002/32/EC of the European Parliament and of the Council of 7 May 2002 on undesirable substances in animal feed—Council statement. L 140, 10–22.

European Commission. (2006a). Commission Recommendation 2006/576/EC of 17 August 2006 on the presence of deoxynivalenol, zearalenone, ochratoxin A, T-2 and HT-2 toxin and fumonisins in products intended for animal feeding. L 229, 7–9.

European Commission. (2006b). Commission Recommendation of 17 August 2006 on the prevention and reduction of Fusarium toxins in cereals and cereal products. L 234, 35–40.
